# HAM/TSP-derived HTLV-1-infected T cell lines promote morphological and functional changes in human astrocytes cell lines: possible role in the enhanced T cells recruitment into Central Nervous System

**DOI:** 10.1186/s12985-015-0398-x

**Published:** 2015-10-12

**Authors:** Eduardo Samo Gudo, Suse Dayse Silva-Barbosa, Leandra Linhares-Lacerda, Marcelo Ribeiro-Alves, Suzana Corte Real, Dumith Chequer Bou-Habib, Wilson Savino

**Affiliations:** National Institute of Health, Ministry of Health, Av. Eduardo Mondlane, 1008 Maputo, Mozambique; Laboratory on Thymus Research, Oswaldo Cruz Institute, Oswaldo Cruz Foundation, Rio de Janeiro, Brazil; National Cancer Institute, Rio de Janeiro, Brazil; Laboratory of Structural Biology, Oswaldo Cruz Institute, Oswaldo Cruz Foundation, Rio de Janeiro, Brazil

**Keywords:** Astrocytes, HTLV-1-infected lymphocytes, Extracellular matrix, Chemokines, Integrins

## Abstract

**Background:**

The mechanisms through which HTLV-1 leads to and maintains damage in the central nervous system of patients undergoing HTLV-1 associated myelopathy/tropical spastic paraparesis (HAM/TSP) are still poorly understood. In recent years, increasing evidence indicates that, not only lymphocytes but also glial cells, in particular astrocytes, play a role in the pathophysiology of HAM/TSP. In this study we used a model of co-culture between human HTLV-1-infected (CIB and C91PL) and non-infected (CEM) T lymphocyte cell lines and astrocyte (U251 and U87) cell lines to mimic the *in vivo* T cell-astrocyte interactions.

**Results:**

We first observed that CIB and C91PL adhere strongly to cultured astrocytes cell lines, and that co-cultures of HTLV-1 infected and astrocyte cell lines cells resulted in rapid syncytium formation, accompanied by severe morphological alterations and increased apoptotic cell death of astrocyte cells. Additionally, cultures of astrocyte cell lines in presence of supernatants harvested from HTLV-1-infected T cell cultures resulted in significant increase in the mRNA of CCL2, CXCL1, CXCL2, CXCL3, CXCL10, IL-13, IL-8, NFKB1, TLR4, TNF, MMP8 and VCAM1, as compared with the values obtained when we applied supernatants of non-infected T- cell lines. Lastly, soluble factors secreted by cultured astrocytic cell lines primed through 1-h interaction with infected T cell lines, further enhanced migratory responses, as compared to the effect seen when supernatants from astrocytic cell lines were primed with non-infected T cell lines.

**Conclusion:**

Collectively, our results show that HTLV-1 infected T lymphocyte cell lines interact strongly with astrocyte cell lines, leading to astrocyte damage and increased secretion of attracting cytokines, which in turn may participate in the further attraction of HTLV-1-infected T cells into central nervous system (CNS), thus amplifying and prolonging the immune damage of CNS.

**Electronic supplementary material:**

The online version of this article (doi:10.1186/s12985-015-0398-x) contains supplementary material, which is available to authorized users.

## Background

Human T-cell leukemia virus type 1 (HTLV-1) is the etiological agent of HTLV-1-associated myelopathy/tropical spastic paraparesis (HAM/TSP), a chronic and slowly progressive neurodegenerative disease of the central nervous system (CNS) [1, 2] and Adult T-cell leukemia/lymphoma (ATL) [3]. Worldwide, an estimated 20 million people are infected with HTLV-1, thus placing this infection as a serious public health problem. Nevertheless, the majority of infected individuals remain asymptomatic carriers and less than 5 % develop HAM/TSP [4–8].

Histopathological findings of CNS revealed that HAM/TSP affects mostly the lower and middle thoracic spinal cord, with marked degeneration of the corticospinal tracts and demyelination, accompanied by diffuse and symmetrical degeneration of the anterolateral and inner portion of the posterior columns [9]. These findings are consistent with the HAM/TSP patient’s neurological symptoms, including spastic paraplegia of the lower extremities, loss of bladder control, and sexual dysfunction [1, 10–12].

To date, the precise mechanisms by which HTLV-1 promotes these lesions remain poorly understood. Nevertheless, available data indicate that progression to HAM/TSP is characterized by presence of an exaggerated and chronic immune response [13], accompanied by massive infiltration of mononuclear cells into the CNS, hyper secretion of pro-inflammatory cytokines and chemokines [14] and spontaneous proliferation T lymphocytes [13–24]. Although the lesions in the CNS have been primarily attributed to these infiltrating lymphocytes, growing evidence points that glial cells, particularly astrocytes, play a key role in this process [14, 25]. Damage of astrocytes is structurally and functionally deleterious to the CNS, since these cells exert important functions, such as: maintenance of the integrity of the blood–brain barrier (BBB), neural cell survival and control of brain excitability [26–28]. Histopathological findings from *post mortem* tissues revealed that astrocytes from HAM/TSP lesions bear an activated phenotype and produce high amounts of pro-inflammatory cytokines, matrix metalloproteinases (MMPs) and chemokines [14, 29, 30]. Additionally, *in vitro* studies demonstrated that interactions with HTLV-1-infected lymphocytes resulted in morphological changes of astrocytes similarly to those found in *post mortem* [31, 32], being accompanied by metabolic deregulation [33, 34].

However the participation of astrocytes in the pathophysiology of HAM/TSP remains poorly understood, particularly their role in the recruitment and trafficking of peripheral T cells into CNS. In this context, we conducted a study to investigate the morphological and functional alterations exerted by HTLV-1-infected T cell lines upon astrocytoma-derived cell lines. In particular, we used an *in vitro* model of T cell-astrocyte cell lines interaction to approach the potential the impact of HTLV-1-infected T cell lines in the integrity and gene expressing profile of migration-related genes of astrocytic cell lines. We also analyzed the migratory response of HTLV-1-T lymphocyte cell lines under the stimulation of astrocytic cell lines primed with supernatants derived from HTLV-1^+^ T cell lines. Our results indicate that under transient interactions with HTLV-1-infected T cell line cells, astrocytic cell lines undergo major morphological changes, together with modulation in the expression of a variety of cell-migration genes. In turn, such reactive astrocytic cell lines increase migratory responses of HTLV-1-infected lymphocytes, thus suggesting a role of these glial elements in the recruitment of additional T cells into CNS.

## Results

### Increased adhesion of HTLV-1-infected T lymphocyte cell lines onto astrocytoma cell lines

**Fig. 1:**
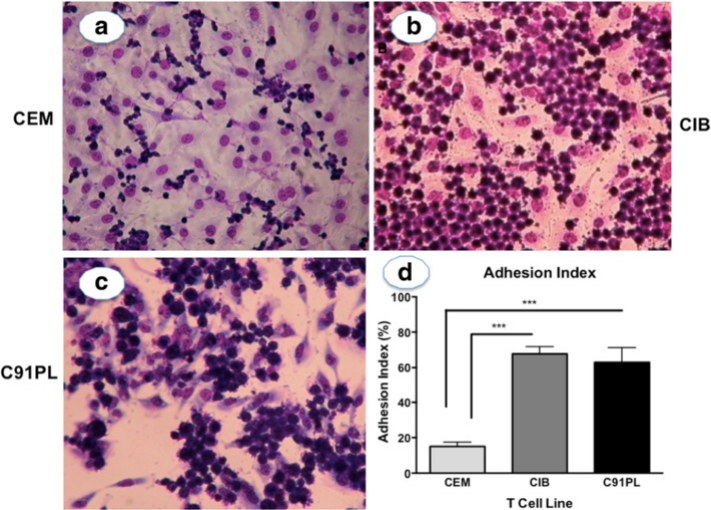
Enhanced adhesion of HTLV-1-infected T cell lines onto human astrocytoma cell lines. HTLV-1-infected (CIB and C91PL) or non-infected (CEM) T cell lines were co-cultured with astrocytoma cell lines (U251) for 30 min. Representative microscopic fields of low magnification indicate higher adhesion degree of HTLV-1-infected T cell lines (**b** and **c**) versus non-infected T cell lines (**a**). Panel d depicts higher adhesion degree of HTLV-1-infected T cell lines (CIB and C91PL) as determined by the measurement of adhesion index. Values in panel (**d**) correspond to mean ± se of 3 independent experiments for each T cell line. *p < 0.05. Original magnifications: ×200

In the first set of experiments, we investigated the adhesion of HTLV-1-infected (CIB and C91PL) and non-infected (CEM) T cell lines to astrocytoma monolayers (U251). The adhesion assay was performed during 30 min, after which non-adherent lymphocytes cell lines were removed and adherent lymphocytes cell lines counted after Giemsa staining. We found that after 30 min in co-cultures, the adhesion degree of HTLV-1 infected T cell lines, (CIB in the Fig. 1b and C91PL in the Fig. 1c) to the astrocytoma cell lines was significantly higher than that of uninfected T cell lines, as illustrated by the measurement of adhesion index of CIB cells (Fig. 1d).

### Co-culture of HTLV-1-T lymphocyte cell lines with astrocytic cell lines results in rapid syncytium formation

**Fig. 2:**
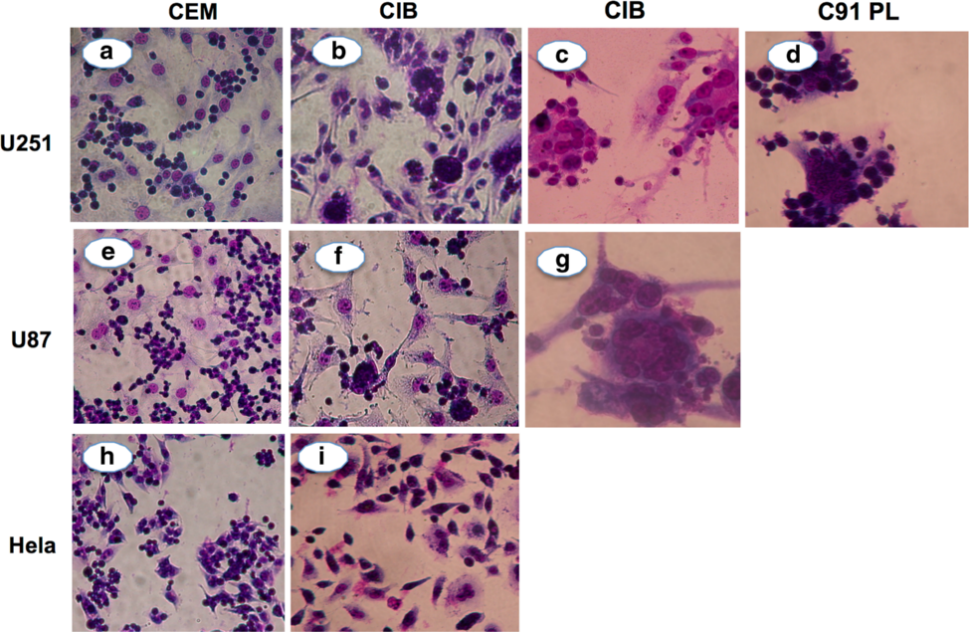
Co-culture of HTLV-1-infected T cell lines with astrocytoma cell lines result in rapid syncytium formation. HTLV-1-infected T cell lines (CIB and C91PL) were co-cultured with astrocyte (U251 and U87) and non-astrocytic (Hela) cell lines for up to 20 h. After 20 h cells were fixed, stained with Giemsa and observed on light microscopy. Syncytia were characterized by presence of large, irregular and multinucleated cells and were observed in panels of co-culture of CIB with U251 (**b**–**c**) and U87 (**f**–**g**) and also in the panels of co-culture of C91PL with U251 (**d**), but not in the co-culture of CEM (**h**) or CIB (**i**) with HeLa cell line. Original magnifications: ×200 (**a–b**, **e–f** and **g–h**) and × 400 (**c–d** and **g**)

Since HTLV-1 infected T cell lines adhered strongly to the astrocytoma monolayers, we investigated the morphological alterations, including syncytium formation after persistent interaction between these cells. For this purpose, HTLV-1 infected T cell lines (CIB and C91PL) were co-cultured with the astrocytoma cell lines (U251 and U87) for up to 20 h, after which, non-adherent cells were removed and the alterations observed after Giemsa staining. We observed heterocellular cell fusion, and syncytium formation between HTLV-1 infected T cell lines and astrocytoma cell lines as early as 6 h post co-culture. These changes were equally seen when the CIB (Fig. 2b, c, f, g) and C91PL (Fig. 2d) HTLV-1 infected cell lines were added onto the astrocytic monolayer. In addition, the effect of HTLV-1 infected cell lines on the astrocytoma cell lines U251 (Fig. 2a–d) and U87 (Fig. 2e–g) was similar. No syncytium could be detected when CEM cells were co-cultured with either astrocytoma monolayer (Fig. 2a, e) or when non astrocytic cell lines (Hela cells) were co-cultured with HTLV-1-infected T cell lines (Fig. 2i).

### Transient contact with HTLV-1-infected cell lines leads to long-term cytopathic effects on astrocytoma cell lines

**Fig. 3:**
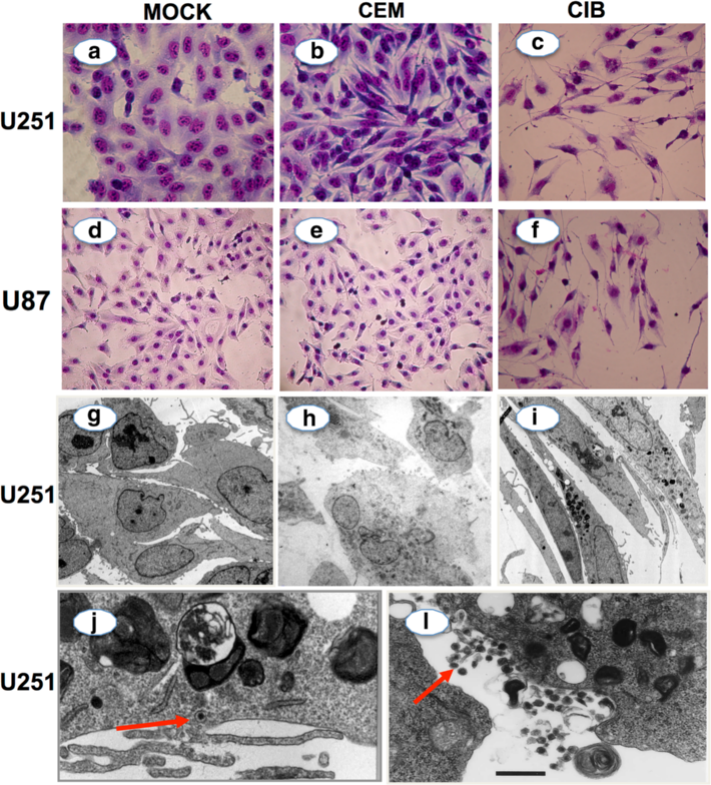
Long-term cellular consequences on astrocytoma cell line monolayers following transient co-culture with HTLV-1-infected cell lines. Long-term effects (5 days post co-culture) exerted by HTLV-1-infected T cell lines on astrocytes cell lines were assessed by co-culture of astrocytoma cell lines (U251 and U87) with HTLV-1-infected (CIB) and non-infected (CEM) T cell lines. Astrocytoma cells were cultured only with RPMI for mock control (**a**, **d** and **g**). Under light microscopy (magnifications = ×200), no morphological alterations were observed when CEM was co-cultured with U251 (**b**) and U87 (**e**). Under light microscopy (magnifications = ×200), morphological alterations on astrocytoma cells were characterized by presence of cell shrinkage, loss of cell-cell contact, seen when CIB cells was co-cultured with U251 (**c**) and U87 cell lines (**f**). Under transmission electron microscopy, ultrastructural alterations were characterized not only by presence of cell shrinkage, loss of cell-cell contact (**i**), but also accumulation of secondary lysosome (**j**), and presence of particles compatible with HTLV-1 virions (highlighted by arrows), seen when U251 astrocytoma cells were co-cultured with CIB lymphocytes (**j**–**l**). No ultrastructural alteration were seen when U251 astrocytoma cells were co-cultured with CEM (**h**)

In the following set of experiments, we were interested to investigate the long term impact of HTLV-1 infected T cells on astrocytes cell lines, including the morphological and ultra structural alterations and induction of apoptosis. For this purpose, we co-cultured astrocytoma cells (U251 and U87) with infected (CIB) or non-infected T cell lines (CEM). After 3 h interaction, lymphocyte cell lines were removed from the co-cultures through vigorous cold PBS washings. Complete removal of T cell lines was confirmed in paired co-cultures stained by Giemsa and observed by light microscopy. The astrocytoma cells were then kept in culture for up to 10 days with daily microscopic observation. Figure 3 shows that 5 days after lymphocyte removal, the astrocytoma cells exhibited important changes characterized by cell shrinkage, loss of cell-cell contact, detachment from the monolayer and cell death (Fig. 3c, f).

These changes were further characterized by transmission electron microscopy (Fig. 3g–i). Astrocytoma cell lines in the monolayers that have been transiently exposed to HTLV-1 (but not to non-infected) T cell lines, exhibited ultrastructural alterations, characterized by severe cell shrinkage, loss of cell-cell contact (Fig. 3i) and accumulation of secondary lysosomes (Fig. 3j). These changes were evident as early as 3 days post washing of T cell lines. In addition, virus-like particles were observed within the cytoplasm of the astrocytoma cells, suggesting that they become infected by HTLV-1 (Fig. 3j, i).

**Fig. 4:**
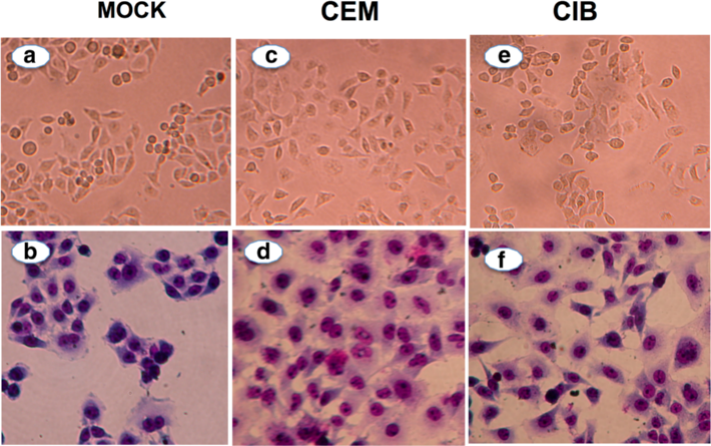
Co-culture of HTLV-1-infected T cell lines with Hela cell line does not yield syncytium formation or morphological alterations. Exposure of Hela cell lines to CEM (**c–d)** or CIB (**e–f**) cell lines does not lead to syncytium formation or morphological alterations. Upper panels show unstained cells observed under optical microscopy and lower panels show Giemsa-stained cells. For Mock controls, cells were cultured with RPMI alone (**a–b**). Cells cultures were maintained for up to 20 h. Original magnifications: ×200

No morphological changes were observed in the mock control (cultures of astrocytoma cells in absence of any T lymphocyte cell lines, Fig. 3a, d and g) and the monolayer previously co-cultured with the non-infected CEM T cell line (Fig. 3b, e, h), as ascertained by Giemsa staining and by electron microscopy. In order to control for nonspecific effects of HTLV-1-infected T cell lines on astrocytoma cell lines, similar experiments were performed using Hela cells, and no morphological alterations were detected in these cells upon interactions with HTLV-1-infected cell lines (Fig. 4).

**Fig. 5:**
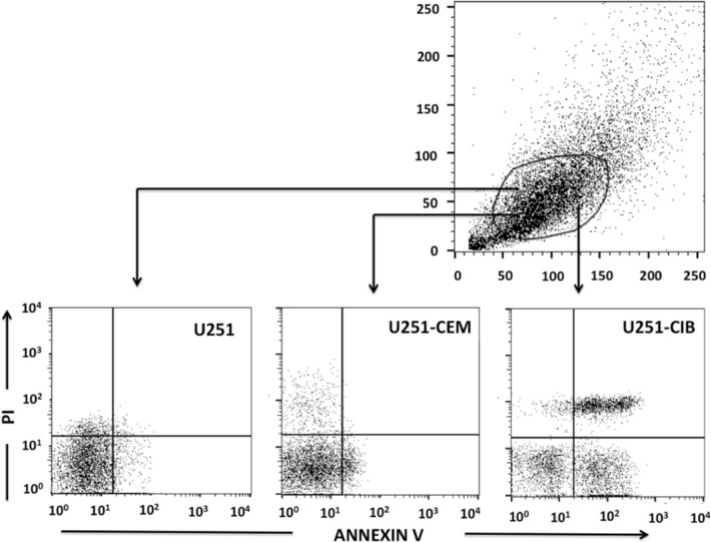
Enhancement of Annexin V binding to astrocytoma cell lines after transient interaction with the HTLV-1-infected T cell lines. Astrocytoma cell lines (U251) were transiently co-cultured with HTLV-1-infected (CIB) and non-infected (CEM) T lymphocyte cell lines. For Mock control, U251 cell were cultured in presence of RPMI alone. Enhanced annexin V binding was observed when U251 was co-cultured with CIB (U215-CIB) as compared to co-culture with CEM cell (U215-CEM) or Mock control (U215)

Subsequent experiments were performed in order to investigate if the cytopathic effect described above involved increased apoptosis. We thus performed annexin V *plus* propidium iodide staining (measured by cytofluorometry) in astrocytoma cell lines after short-term interaction with each T cell line. Figure 5 shows that transient contact with HTLV-1-infected T lymphocyte cell lines resulted in enhanced annexin V labeling (as compared to the values obtained with the non-infected T cell line), suggesting increased apoptosis, as further discriminated from necrotic cell death by the propidium iodide staining profiles.

### Expression pattern of cell adhesion and migration-related genes in astrocytoma cell lines following short-term incubation with supernatants from HTLV-1^+^ T cell lines

**Fig. 6:**
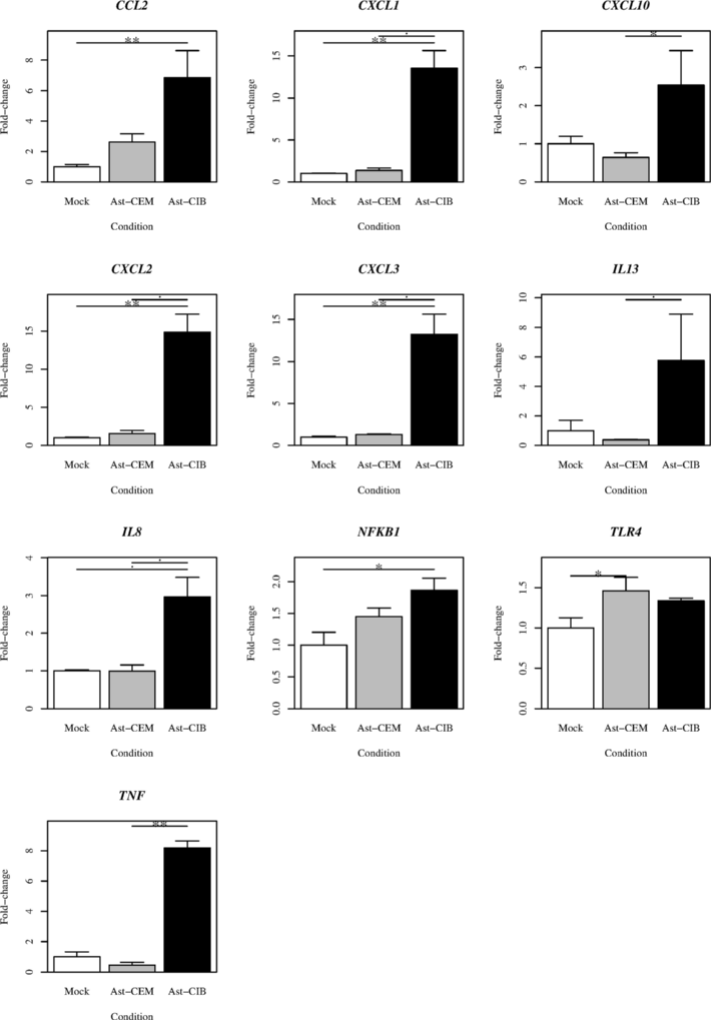
Modulation of cell adhesion and migration-related gene expression in astrocytoma cell lines after transient exposure to supernatants of HTLV-1-infected T cell lines. Astrocytoma cell lines (U251) were primed with supernatants from CIB (Ast-CIB) and CEM (Ast-CEM) T cells cultures. For Mock control, astrocytoma cells were treated with RPMI alone (Mock). After 1 h of exposure, cells were washed, harvested and RNA was extracted. Ast-CIB expressed statistically significant higher amounts of RNA for all illustrated genes as compared to Ast-CEM and Mock. Data derive from 3 independent experiments

Since soluble factors secreted by HTLV-1 infected T cell lines, particularly soluble Tax1 protein, have been described to exert paracrine effect on target cells [15], we investigated whether supernatants from HTLV-1^+^ T cell lines could affect the expression of cell adhesion and migration-related genes on astrocytoma cell lines. Indeed, transient exposure (30 min) to fractionated supernatants from the HTLV-1^+^ cell lines resulted in statistically significant increase in the mRNA expression of various cytokines/chemokines and adhesion molecules, including the pro-inflammatory cytokine TNF-α, the TNF-α-regulated chemokines CXCL1, CXCL2, CXCL3, CXCL8, CCL2, IL13 and CXCL10, as well as V-CAM-1 and MMP-8, as compared to the values obtained when the astrocytoma cells were subjected to supernatants from non-infected T cell line cultures (Fig. 6).

Of note, although not statistically significant, qPCR values for gene expression for various other chemokines and ECM/adhesion molecules were slightly higher (Additional file 1: Figure S1A–D and Additional file 2: Figure S2A–D).

### Enhanced migration of HTLV-1-infected T cell lines after stimulation with supernatants from primed astrocytoma cell lines

**Fig. 7:**
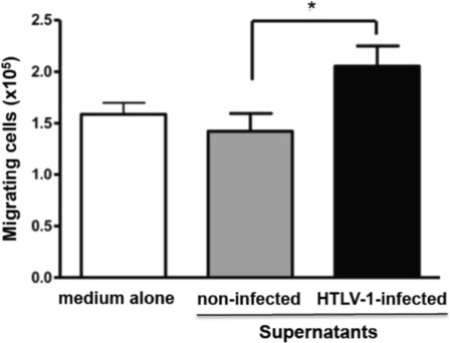
Enhanced migratory response of HTLV-1-infected T lymphocyte cell lines treated with supernatants from astrocytoma cell lines primed after transient interaction with HTLV-1-infected T cell lines. The HTLV-1-infected T cell line (CIB) was allowed to migrate in transwell chambers whose bottom wells contained supernatants from astrocytoma cell cultures that were primed with HTLV-1-infected versus non-infected T cell lines. Migrating cells, harvested in the lower chambers, were counted using trypan blue cell viability analysis. Values correspond to mean ± standard error of 3 independent experiments for each T cell line. *p < 0.05

Considering that the expression pattern of various cell migration-related genes was up-regulated in the astrocytoma cell lines cultured in presence of supernatants from HTLV-1^+^ T cell lines, it was plausible to hypothesize that, once astrocytes are primed by a first wave of infiltrating T cells, they might play a role in the further recruitment of T cells into the central nervous system. In order to address this issue we applied supernatants from astrocytoma cell cultures (U251) previously primed with a transient exposure with the HTLV-1 infected (CIB) or non-infected (CEM) T cell lines, as migratory *stimuli* for the migration of further HTLV-1^+^ T cells. Migratory response of these cells to supernatant of astrocytoma cell lines primed with infected cell lines was significantly enhanced, as compared to supernatants from untreated astrocytoma cell lines, or those co-cultured with the non-infected T cell line (Fig. 7).

## Discussion

The mechanisms underlying the pathogenesis of HAM/TSP are still poorly understood. Although previous evidence strongly suggests that astrocytes take part in this process through several mechanisms [30–32], their participation in the migration of T cells into the CNS has not been studied in deep. Given the central role of astrocytes in the functional and structural homeostasis of the CNS [28], any impairment of these cells would potentially compromise CNS functional and structural integrity. A better understanding of these processes is thus essential for designing future effective therapeutic and preventive strategies. In this context, we investigated the effects exerted by HTLV-1 on astrocytes using an *in vitro* model of heterocellular interaction involving T cell lines and astrocytoma cell. Experimentally, we used a T cell line derived from a HAM/TSP patient (CIB), a T cell line originally infected by HTLV-1 *in vitro* (C91PL) and two astrocytoma cell lines (U251 and U87).

We first demonstrated that HTLV-1-infected T cell lines adhered significantly higher to the astrocytoma monolayers as compared to the non-infected T cell line. This finding suggests that astrocytes may represent a critical target of HTLV-1 infected lymphocytes. Previous studies also demonstrated that HTLV-1 infected cell lines adhered stronger to endothelial cell lines [35]. We also observed that co-culture of astrocytoma cell lines with HTLV-1 infected, (but not with non-infected) T cell lines, resulted in rapid syncytium formation, seen as early as 6 h post co-culture. In addition, no syncytium was observed when a non-astrocyte cell line was used as a control, demonstrating that this effect is at least to some extent cell type specific. This result is in agreement with previous observations, in which rapid syncytium formation was detected when HTLV-1 infected lymphocyte cell lines were co-cultured with astrocyte cell lines [32]. These findings allow us to hypothesize that HTLV-1 infected cells have an increased ability to cross the blood–brain barrier into the CNS, where they rapidly and strongly bind to the astrocytoma, leading to severe functional and morphological damage.

In a second set of experiments, we observed several and extensive morphological changes and cytopathic effect in 5-day culture of astrocytoma cell cultures that were transiently exposed to HTLV-1-infected T cell lines, but not to non-infected counterparts. The cytopathic effect was characterized by loss of cell-cell contact, cell shrinkage, accumulation of lysosomal vesicles and cell death. Notably, similar morphological alterations have been observed in endothelial cells after transient interaction with the MT-2 HTLV-1^+^ T cell line [35]. These alterations were rather cell type specific since co-culture of HTLV-1 infected T cell lines with the Hela cell line, a non-astrocytic lineage, did not result in any cytopathic effect (see Fig. 4). We also detected an enhancement of astrocytoma apoptosis secondary to the interaction with HTLV-1-infected cells as shown in Fig. 5, a finding corroborated by similar results previously reported [36–38]. Several mechanisms has been proposed to explain the apoptotic effect of HTLV-1 infected T cells on astrocytes, such as the direct effect of secreted Tax-1 protein in inducing apoptotic pathways in the target cell through the down regulation in the cellular expression of *bcl-2* [36, 37] and susceptibility of astrocytes to apoptosis secondary to increase in the production of TNF-α [38].

Of note, we observed the presence of HTLV-1 viral particles in the cytoplasm of astrocytoma cells after transient interaction with HTLV-1^+^ T cells line, but not with the non-infected T cell line. Infection of astrocytes with HTLV-1 has been reported in previous studies [39–41], and suggests that direct infection may also be involved in the functional and morphological damage of human astrocytes in HAM/TSP.

We also investigated the role played by the soluble factors secreted by HTLV-1^+^ T cell lines on cultured astrocyte cell lines. We used qPCR arrays to quantitatively evaluate the expression levels of a large numbers of cell migration-related genes, including cytokines/chemokines and extracellular matrix/cell adhesion proteins. A short-term (1 h) exposure of astrocytoma cell lines to fractionated HTLV-1-infected T cell lines-derived supernatants, resulted in statistically significant increase in the mRNA of TNF-α, various chemokines, as well as VCAM-1 and MMP-8, as compared with supernatants derived from the non-infected T cell line, as shown in Fig. 6. These results strongly corroborate the findings reported by Ando and cols. (2013), who found an increased CXCL10 production by astrocytes that were transiently co-cultured with CIB cells [42] and also with results reported by Tomoo and cols. (2013), who found that level of CXCL10 in the CSF correlated with progression to HAM/TSP [43], suggesting that CXCL10 might play a critical role in stimulating migration of HTLV-1 infected T cells in the transwell chamber and ultimately reflecting what happens *in vivo*, when T-cells migrate into the CNS. Our results are also in line with previous histopathological findings from post-mortem tissues, in which it has been found that astrocytes from HAM/TSP lesions exhibit higher contents of proinflammatory cytokines, MMP and chemokines [14, 29, 30].

Moreover, our data corroborate those described by Akaoka (2011) and Szymocha (2000), who demonstrated that infiltrating HTLV-1-infected T lymphocytes in the CNS continuously induce significant changes in astrocytes, not only through cell-cell interaction [44], but also through soluble elements, particularly the 42 kDa Tax-1 viral protein [31]. Tax is a transactivator/oncoprotein thought to play an important role in the course of HAM/TSP, and soluble Tax1 has been shown to induce functional changes in human astrocytes [36] and to modulate gene expression on target cells [15].

Conjointly, these findings strongly point to an important paracrine effect of HTLV-1 infected lymphocytes on astrocytes and highlight that the functional impairment of astrocytes occurring in HAM/TSP, would be much more complex than ever described.

Our findings suggest that the functional impairment of astrocytes caused by interactions with HTLV-1-infected T cells contributes to the perpetuation and amplification of the CNS damage. Considering that we applied a 30–50 kDa supernatant fraction to stimulate the astrocytoma cells, it is conceivable that Tax1 protein is involved in these effects. Yet, additional studies with blocking antibodies are required to confirm this hypothesis.

Since astrocytes primed with HTVL-1-infectd T cells expressed higher levels of various cell adhesion and migration-related mRNA molecules, it seemed plausible to hypothesize that supernatants from such primary cultures could enhance T cell migration. Actually, this was the case and supernatants from HTLV-1 primed astrocytoma cell lines were able increase the migration of HTLV-1 infected T cell lines. Conceptually, these data suggest that activated astrocytes would enhance the recruitment of additional T cells into the CNS. Since main focus of this study was to understand the impact of a HAM/TSP derived T cell line (CIB), the *in vitro* derived HTLV-1 infected T cell line (C91PL) was used as a control only in the first set of experiments when we observed that the effects were similar when using both cell lines.

Although we have not performed immune staining to confirm the presence of HTLV-1 inside the cells, we believe that the viral particle detection by electron microscopy is a convincing evidence of astrocyte infection. Similarly, we also think that the enhanced T cell migration after being primed by supernatants from infected astrocytes suggests an increased concentration of chemotactic factors, as indicated by the elevated expression of the corresponding mRNAs by HTLV-1-infected astrocytes.

Of remark, our results are of utmost importance, since most of the previous work that investigated HTLV-1-induced changes on astrocytes used *in vitro* derived HTLV-1-infected T cell lines, such as MT-2 or C91PL [29, 31–34, 45]. In our study we used a HAM/TSP patient-derived lymphocyte T cell line. Indeed, to our knowledge, only Ando and co-workers conducted functional assays on astrocytes using same cell line [42].

## Conclusion

In conclusion, our results show that HAM/TSP-derived T lymphocyte cell lines exert a pleiotropic effects upon astrocyte cell lines, leading to astrocyte damage and increased secretion of attracting cytokines, which in turn may participate in the further attraction of HTLV-1-infected T cells into central nervous system (CNS), thus amplifying and prolonging the immune damage of CNS.

## Methods

### Cell lines

The human malignant astrocytoma cell lines U251 and U87, both derived from malignant gliobastoma multiforme (gently provided by Dr Maria do Socorro Pombo de Oliveira, National Cancer Institute, Rio de Janeiro) [46], were grown in high glucose (4500 mg/l) Dulbecco Modified Eagle Medium (DMEM; Gibco-BRL, Grand Island, NY) supplemented with 10 % fetal calf serum (FCS) (Cultilab, Campinas, Brasil), 2 mM L-glutamine (Gibco, Scotland, UK), 100 U/ml penicillin and 100 g/ml streptomycin (Gibco, Scotland, UK) at 37 °C in a humidified incubator with 5 % CO_2_. Hela cells, an epithelial cell line derived from cervical cancer cells [47], were used as a negative control in some experiments. The HTLV-1-infected T cell line CIB (gently provided by Dr. Olivier Hermine, Necker Hospital, Paris) was derived from a HAM/TSP patient [48]. The HTLV-1-infected T cell line C91PL was derived from human cord blood T lymphocytes immortalized by HTLV-I infection [49] (used in several assays as a positive control and gently provided by Dr. Olivier Hermine, Necker Hospital, Paris). The non-infected T-cell line CEM, derived from a patient with acute lymphoblastic leukemia, [50] (used as a negative control and gently provided by Dr Maria do Socorro Pombo de Oliveira, National Cancer Institute, Rio de Janeiro) was cultured in RPMI 1640 supplemented with 10 % fetal calf serum, 100 U/ml penicillin and 100 g/ml streptomycin at 37 °C, 5 % CO_2_. Cultures of the HTLV-1-infected T cell lines were further supplemented with 100 U/ml of interleukin 2 (IL-2) (Roche, France).

### Heterocellular adhesion assay

For adhesion assays, astrocytoma and Hela cells were first plated in 25 cm^2^ flasks until sub-confluence. Then, HTLV-1-infected or non-infected T lymphocytes were added to the astrocyte monolayer at a ratio astrocyte: lymphocyte of 1:4 and allowed to adhere for 30 min at 37 °C, 5 % CO_2_ in RPMI 1640 supplemented with 2 mM L-glutamine and antibiotics in absence of FCS and IL-2. Supernatants containing floating lymphocytes were then discarded and the contents of each flask (astrocytes + adhered lymphocytes) were fixed in absolute ethanol, stained with Giemsa and counted to determine the adhesion index (AI), as previously described [51] and validated for this type of analysis [52], using the following formula:

$$ \mathrm{AI} = \frac{\mathrm{Astrocytes}\ \mathrm{with}\ \mathrm{adhered}\ \mathrm{lymphocytes}}{\mathrm{Total}\ \mathrm{number}\ \mathrm{o}\mathrm{f}\ \mathrm{astrocytes}}\times \frac{\mathrm{Lymphocytes}\ \mathrm{adhered}\ \mathrm{t}\mathrm{o}\ \mathrm{astrocytes}}{\mathrm{Total}\ \mathrm{number}\ \mathrm{o}\mathrm{f}\ \mathrm{astrocytes}}\times 100 $$

### Assessment of syncytium formation

To assess syncytium formation, astrocytoma cells were initially grown in 25 cm^2^ flasks until sub-confluence. HTLV-1-infected T lymphocytes (CIB or C91PL) or the non-infected T cell line (CEM) were added to the monolayer of astrocytoma at astrocyte/lymphocyte ratio of 1:4 and left to adhere for up to 20 h in culture conditions similar to those described above. Supernatant was then discarded, washed briefly with PBS, fixed in absolute ethanol, stained with Giemsa and observed by light microscopy. Similar assays were performed using a non-astrocytic cell line (Hela), to assess whether changes were specific for astrocytes.

### Morphological assessment of cultured astrocytoma cells

Astrocytoma cells were initially grown in 25 cm^2^ flasks until sub-confluence. Transient co-cultures of astrocytes with either HTLV-1-infected T lymphocytes (CIB or C91PL) or the non-infected T cell line (CEM) were performed by replacing the medium in the astrocytoma cultures with those from cultures of HTLV-1-infected or non-infected T cell lines in the absence of IL-2 at 37 °C, 5 % CO_2_, at astrocyte/lymphocyte ratio of 1:4. After 3 h, lymphocytes were completely removed from the cultures by vigorous washing using cold PBS. Duration of co-culture was set at 3 h, since the study conducted by Mor-Vaknin et al., [32], demonstrated that syncytium was formed as early as 4 h post co-culture and in our experiments syncytium was formed as early as 6 h post co-culture.

Hela cells were used as controls for nonspecific interactions between astrocytes and HTLV-1-infected cells. For Mock controls, astrocytes were culture alone with RPMI.

After lymphocyte removal by vigorous washings with cold PBS, astrocytoma and Hela cells were kept in culture for up to 5 days, replenishing fresh medium every two days. At days 5 post co-culture, astrocytes were processed for optical or transmission electron microscopy, as previously described [53], using current 2.5 % glutaraldehyde 1 % paraformaldehyde fixation, followed by uranyl acetate plus lead citrate staining, with the analysis of ultrathin sections being done under a transmission electron microscope (Jeol JEM-1011) on the Rudolf Barth Electron Microscopy Platform at the Oswaldo Cruz Institute (Fiocruz, Rio de Janeiro).

### Apoptosis of astrocytoma cells induced by HTLV-1-infected lymphocytes

The ability of HTLV-1 or non-infected T cells to induce apoptosis on the astrocytoma cells was measured by cytofluorometry using FITC-coupled Annexin V Apoptosis Detection Kit I (Becton-Dickinson/Pharmingen, San Diego). After transient co-cultures during 3 h, followed by lymphocyte removal by vigorous washings with cold PBS, astrocytes were kept in culture for additional 2 h, trypsinized and stained with FITC-Annexin V and Propidium Iodide according to the manufacturer’s instructions. Events were acquired using a FACSCalibur Flow Cytometer (Becton-Dickinson, San Diego, USA).

### Cell adhesion and cell migration-related gene expression of astrocytoma cells primed with supernatants from HTLV-1-infected T lymphocytes

We further evaluated the effect exerted by HTLV-1-infected cells on the expression of cell adhesion and cell migration-related genes in astrocytoma cells. Astrocytes were primed using supernatants from HTLV-1-infected T cell cultures, using as control counterpart supernatants from non-infected T cell cultures. For this purpose, lymphocytes were grown in 75 cm^2^ culture flasks at an initial concentration of 1x10^7^ cells/mL during 24hs. Supernatants were then collected and concentrated using the centripep YM-50 and YM-30 filters (Millipore, corporation, County Cork, Ireland), according to the manufacturer’s instructions. The fraction was added to the astrocyte monolayer for 1 h at 37 °C and then, cells were then trypsinized and washed. As a mock control, growing astrocytes were treated with RPMI alone.

Total RNA was extracted using RNeasy Micro Kit (Qiagen, Hilden, Germany), according to the manufacturers instructions, including DNAse treatment. Total RNA was quantified by spectrophotometry using Nanodrop 2000 (Thermo Scientific, USA). RNA integrity was assessed for the presence of ribosomal RNA 28S and 18S, using denaturating agarose gel electrophoresis as previously described [54]. RNA was used for the RT-PCR if the ratio A_260_/A_230_ ≈ 2.0.

Gene expressing profiles for cytokines/chemokines and extracellular matrix (ECM) proteins/adhesion molecules were ascertained by quantitative PCR. cDNAs were prepared from 1 μg RNA of each sample using “RT^2^ First Strand” (SABioscience, Maryland, USA), according to the manufacturers instructions. Then, RT^2^ SYBR Green/ROX PCR Master Mix (SABioscience) and’ nuclease-free water were added to the cDNA. Twenty-five μL of this mixture were added to 96 microplates containing primers for each of the 84 target genes for ECM proteins/adhesion molecules using corresponding qPCR arrays (SABioscience/Qiagen, USA), as seen in supplementary data in S1 Table and S2 Table. Amplification signals were captured using the ABI Prism 7000 PCR device (Applied Biosystems, Foster City, CA, USA). Melting curve analysis was performed for each sample at the end of the PCR.

### Migration profile of HTLV-1-T lymphocytes cell lines under the stimulation of supernatants from primed astrocytoma cell lines

Cell migration studies were conducted in *transwell* chambers (Corning Costar, Cambridge, MA, USA), as described elsewhere [55], using the 8-μm pore size inserts coated with 10 μg/ml of BSA, for 1 h at 37 °C. Supernatants from astrocytoma cultures previously primed with HTLV-1-T cell lines **(**3 h co-culture**)** were harvested and added into the lower chamber, whereas 10^6^ CIB cells/100 μl (of RPMI/1 % BSA) were added into the upper chambers. After 4 h incubation at 37 °C in 5 % CO_2_, we counted the cells that migrated into the lower chambers using trypan blue cell viability analysis.

### Statistical analysis

All statistical analyses were performed using the GraphPad Prism 5.01 software package. Quantitative data were expressed as the mean ± standard error. The Student’s *t*-test and the One Way ANOVA test were used to compare the differences among groups. Differences were considered statistically significant when the p values were ≤ 0.05.

## Additional files

Additional file 1: Figure S1. Cytokine/Chemokine gene expression in astrocytoma cell line after transient exposure to supernatants of HTLV-1-infected T cell lines. The astrocyte cell line (U251) was primed with supernatants from cultured CIB (Ast-CIB) and CEM (Ast-CEM) T cells. For Mock control, astrocytoma cells were treated with RPMI alone (Mock). After 1 h of exposure, cells were washed, harvested and RNA was extracted. Ast-CIB expressed statistically significant higher amounts of RNA for all illustrated genes as compared to Ast-CEM and Mock. Data derive from 3 independent experiments. (PDF 614 kb) Additional file 2: Figure S2. Extracellular matrix proteins gene expression in astrocytoma cell lines after transient exposure to supernatants of HTLV-1-infected T cell lines. The astrocytoma (U251) were primed with supernatants from cultured CIB (Ast-CIB) and CEM (Ast-CEM) T cell lines. For Mock control, astrocytoma cells were treated with RPMI alone (Mock). After 1 h of exposure, cells were washed, harvested and RNA was extracted. Ast-CIB expressed statistically significant higher amounts of RNA for all illustrated genes as compared to Ast-CEM and Mock. Data derive from 3 independent experiments. (PDF 592 kb)
