# Supplementary material for: HAM/TSP-derived HTLV-1-infected T cell lines promote morphological and functional changes in human astrocytes cell lines: possible role in the enhanced T cells recruitment into Central Nervous System
Source: Virol J. 2015 Oct 12;12:165. doi: 10.1186/s12985-015-0398-x (PMC4603815; doi:10.1186/s12985-015-0398-x)
Supplement: Additional file 1: Figure S1. — Cytokine/Chemokine gene expression in astrocytoma cell line after transient exposure to supernatants of HTLV-1-infected T cell lines. The astrocyte cell line (U251) was primed with supernatants from cultured CIB (Ast-CIB) and CEM (Ast-CEM) T cells. For Mock control, astrocytoma cells were treated with RPMI alone (Mock). After 1 h of exposure, cells were washed, harvested and RNA was extracted. Ast-CIB expressed statistically significant higher amounts of RNA for all illustrated genes as compared to Ast-CEM and Mock. Data derive from 3 independent experiments. (PDF 614 kb) [file 12985_2015_398_MOESM1_ESM.docx]

**S1 Table.** Genes of the Cytokine/Chemokine PCR Array

| **Symbol** | **Description** |
| --- | --- |
| ADIPOQ | Adiponectin, C1Q and collagen domain containing |
| BMP2 | Bone morphogenetic protein 2 |
| BMP4 | Bone morphogenetic protein 4 |
| BMP6 | Bone morphogenetic protein 6 |
| BMP7 | Bone morphogenetic protein 7 |
| C5 | Complement component 5 |
| CCL1 | Chemokine (C-C motif) ligand 1 |
| CCL11 | Chemokine (C-C motif) ligand 11 |
| CCL13 | Chemokine (C-C motif) ligand 13 |
| CCL17 | Chemokine (C-C motif) ligand 17 |
| CCL18 | Chemokine (C-C motif) ligand 18 (pulmonary and activation-regulated) |
| CCL19 | Chemokine (C-C motif) ligand 19 |
| CCL2 | Chemokine (C-C motif) ligand 2 |
| CCL20 | Chemokine (C-C motif) ligand 20 |
| CCL21 | Chemokine (C-C motif) ligand 21 |
| CCL22 | Chemokine (C-C motif) ligand 22 |
| CCL24 | Chemokine (C-C motif) ligand 24 |
| CCL3 | Chemokine (C-C motif) ligand 3 |
| CCL5 | Chemokine (C-C motif) ligand 5 |
| CCL7 | Chemokine (C-C motif) ligand 7 |
| CCL8 | Chemokine (C-C motif) ligand 8 |
| CD40LG | CD40 ligand |
| CNTF | Ciliary neurotrophic fator |
| CSF1 | Colony stimulating factor 1 (macrophage) |
| CSF2 | Colony stimulating factor 2 (granulocyte-macrophage) |
| CSF3 | Colony stimulating factor 3 (granulocyte) |
| CX3CL1 | Chemokine (C-X3-C motif) ligand 1 |
| CXCL1 | Chemokine (C-X-C motif) ligand 1 (melanoma growth stimulating activity, alpha) |
| CXCL10 | Chemokine (C-X-C motif) ligand 10 |
| CXCL11 | Chemokine (C-X-C motif) ligand 11 |
| CXCL12 | Chemokine (C-X-C motif) ligand 12 |
| CXCL13 | Chemokine (C-X-C motif) ligand 13 |
| CXCL16 | Chemokine (C-X-C motif) ligand 16 |
| CXCL2 | Chemokine (C-X-C motif) ligand 2 |
| CXCL5 | Chemokine (C-X-C motif) ligand 5 |
| CXCL9 | Chemokine (C-X-C motif) ligand 9 |
| FASLG | Fas ligand (TNF superfamily, member 6) |
| GPI | Glucose-6-phosphate isomerase |
| IFNA2 | Interferon, alpha 2 |
| IFNG | Interferon, gamma |
| IL10 | Interleukin 10 |
| IL11 | Interleukin 11 |
| IL12A | Interleukin 12A (natural killer cell stimulatory factor 1, cytotoxic lymphocyte maturation factor 1, p35) |
| IL12B | Interleukin 12B (natural killer cell stimulatory factor 2, cytotoxic lymphocyte maturation factor 2, p40) |
| IL13 | Interleukin 13 |
| IL15 | Interleukin 15 |
| IL16 | Interleukin 16 |
| IL17A | Interleukin 17A |
| IL17F | Interleukin 17F |
| IL18 | Interleukin 18 (interferon-gamma-inducing factor) |
| IL1A | Interleukin 1, alpha |
| IL1B | Interleukin 1, beta |
| IL1RN | Interleukin 1 receptor antagonist |
| IL2 | Interleukin 2 |
| IL21 | Interleukin 21 |
| IL22 | Interleukin 22 |
| IL23A | Interleukin 23, alpha subunit p19 |
| IL24 | Interleukin 24 |
| IL27 | Interleukin 27 |
| IL3 | Interleukin 3 (colony-stimulating factor, multiple) |
| IL4 | Interleukin 4 |
| IL5 | Interleukin 5 (colony-stimulating factor, eosinophil) |
| IL6 | Interleukin 6 (interferon, beta 2) |
| IL7 | Interleukin 7 |
| IL8 | Interleukin 8 |
| IL9 | Interleukin 9 |
| LIF | Leukemia inhibitory factor (cholinergic differentiation factor) |
| LTA | Lymphotoxin alpha (TNF superfamily, member 1) |
| LTB | Lymphotoxin beta (TNF superfamily, member 3) |
| MIF | Macrophage migration inhibitory factor (glycosylation-inhibiting factor) |
| MSTN | Myostatin |
| NODAL | Nodal homolog (mouse) |
| OSM | Oncostatin M |
| PPBP | Pro-platelet basic protein (chemokine (C-X-C motif) ligand 7) |
| SPP1 | Secreted phosphoprotein 1 |
| TGFB2 | Transforming growth factor, beta 2 |
| THPO | Thrombopoietin |
| TNF | Tumor necrosis factor |
| TNFRSF11B | Tumor necrosis factor receptor superfamily, member 11b |
| TNFSF10 | Tumor necrosis factor (ligand) superfamily, member 10 |
| TNFSF11 | Tumor necrosis factor (ligand) superfamily, member 11 |
| TNFSF13B | Tumor necrosis factor (ligand) superfamily, member 13b |
| VEGFA | Vascular endothelial growth factor A |
| XCL1 | Chemokine (C motif) ligand 1 |
