# Supplementary material for: HAM/TSP-derived HTLV-1-infected T cell lines promote morphological and functional changes in human astrocytes cell lines: possible role in the enhanced T cells recruitment into Central Nervous System
Source: Virol J. 2015 Oct 12;12:165. doi: 10.1186/s12985-015-0398-x (PMC4603815; doi:10.1186/s12985-015-0398-x)
Supplement: Additional file 2: Figure S2. — Extracellular matrix proteins gene expression in astrocytoma cell lines after transient exposure to supernatants of HTLV-1-infected T cell lines. The astrocytoma (U251) were primed with supernatants from cultured CIB (Ast-CIB) and CEM (Ast-CEM) T cell lines. For Mock control, astrocytoma cells were treated with RPMI alone (Mock). After 1 h of exposure, cells were washed, harvested and RNA was extracted. Ast-CIB expressed statistically significant higher amounts of RNA for all illustrated genes as compared to Ast-CEM and Mock. Data derive from 3 independent experiments. (PDF 592 kb) [file 12985_2015_398_MOESM2_ESM.docx]

**S2 Table.** Genes screened in the extracellular matrix-Adhesion Molecule PCR Array

| **Symbol** | **Description** |
| --- | --- |
| ADAMTS1 | ADAM metallopeptidase with thrombospondin type 1 motif, 1 |
| ADAMTS13 | ADAM metallopeptidase with thrombospondin type 1 motif, 13 |
| ADAMTS8 | ADAM metallopeptidase with thrombospondin type 1 motif, 8 |
| CD44 | CD44 proteoglycan (Indian blood group) |
| CDH1 | Cadherin 1, type 1, E-cadherin (epithelial) |
| CNTN1 | Contactin 1 |
| COL11A1 | Collagen, type XI, alpha 1 |
| COL12A1 | Collagen, type XII, alpha 1 |
| COL14A1 | Collagen, type XIV, alpha 1 |
| COL15A1 | Collagen, type XV, alpha 1 |
| COL16A1 | Collagen, type XVI, alpha 1 |
| COL1A1 | Collagen, type I, alpha 1 |
| COL4A2 | Collagen, type IV, alpha 2 |
| COL5A1 | Collagen, type V, alpha 1 |
| COL6A1 | Collagen, type VI, alpha 1 |
| COL6A2 | Collagen, type VI, alpha 2 |
| COL7A1 | Collagen, type VII, alpha 1 |
| COL8A1 | Collagen, type VIII, alpha 1 |
| VCAN | Versican |
| CTGF | Connective tissue growth factor |
| CTNNA1 | Catenin (cadherin-associated protein), alpha 1, 102kDa |
| CTNNB1 | Catenin (cadherin-associated protein), beta 1, 88kDa |
| CTNND1 | Catenin (cadherin-associated protein), delta 1 |
| CTNND2 | Catenin (cadherin-associated protein), delta 2 (neural plakophilin-related arm-repeat protein) |
| ECM1 | Extracellular matrix protein 1 |
| FN1 | Fibronectin 1 |
| HAS1 | Hyaluronan synthase 1 |
| ICAM1 | Intercellular adhesion molecule 1 |
| ITGA1 | Integrin, alpha 1 |
| ITGA2 | Integrin, alpha 2 (CD49B, alpha 2 subunit of VLA-2 receptor) |
| ITGA3 | Integrin, alpha 3 (antigen CD49C, alpha 3 subunit of VLA-3 receptor) |
| ITGA4 | Integrin, alpha 4 (antigen CD49D, alpha 4 subunit of VLA-4 receptor) |
| ITGA5 | Integrin, alpha 5 (fibronectin receptor, alpha polypeptide) |
| ITGA6 | Integrin, alpha 6 |
| ITGA7 | Integrin, alpha 7 |
| ITGA8 | Integrin, alpha 8 |
| ITGAL | Integrin, alpha L (antigen CD11A (p180), lymphocyte function-associated antigen 1; alpha polypeptide) |
| ITGAM | Integrin, alpha M (complement component 3 receptor 3 subunit) |
| ITGAV | Integrin, alpha V (vitronectin receptor, alpha polypeptide, antigen CD51) |
| ITGB1 | Integrin, beta 1 (fibronectin receptor, beta polypeptide, antigen CD29 includes MDF2, MSK12) |
| ITGB2 | Integrin, beta 2 (complement component 3 receptor 3 and 4 subunit) |
| ITGB3 | Integrin, beta 3 (platelet glycoprotein IIIa, antigen CD61) |
| ITGB4 | Integrin, beta 4 |
| ITGB5 | Integrin, beta 5 |
| KAL1 | Kallmann syndrome 1 sequence |
| LAMA1 | Laminin, alpha 1 |
| LAMA2 | Laminin, alpha 2 |
| LAMA3 | Laminin, alpha 3 |
| LAMB1 | Laminin, beta 1 |
| LAMB3 | Laminin, beta 3 |
| LAMC1 | Laminin, gamma 1 (formerly LAMB2) |
| MMP1 | Matrix metallopeptidase 1 (interstitial collagenase) |
| MMP10 | Matrix metallopeptidase 10 (stromelysin 2) |
| MMP11 | Matrix metallopeptidase 11 (stromelysin 3) |
| MMP12 | Matrix metallopeptidase 12 (macrophage elastase) |
| MMP13 | Matrix metallopeptidase 13 (collagenase 3) |
| MMP14 | Matrix metallopeptidase 14 (membrane-inserted) |
| MMP15 | Matrix metallopeptidase 15 (membrane-inserted) |
| MMP16 | Matrix metallopeptidase 16 (membrane-inserted) |
| MMP2 | Matrix metallopeptidase 2 (gelatinase A, 72kDa gelatinase, 72kDa type IV collagenase) |
| MMP3 | Matrix metallopeptidase 3 (stromelysin 1, progelatinase) |
| MMP7 | Matrix metallopeptidase 7 (matrilysin, uterine) |
| MMP8 | Matrix metallopeptidase 8 (neutrophil collagenase) |
| MMP9 | Matrix metallopeptidase 9 (gelatinase B, 92kDa gelatinase, 92kDa type IV collagenase) |
| NCAM1 | Neural cell adhesion molecule 1 |
| PECAM1 | Platelet/endothelial cell adhesion molecule |
| SELE | Selectin E |
| SELL | Selectin L |
| SELP | Selectin P (granule membrane protein 140kDa, antigen CD62) |
| SGCE | Sarcoglycan, epsilon |
| SPARC | Secreted protein, acidic, cysteine-rich (osteonectin) |
| SPG7 | Spastic paraplegia 7 (pure and complicated autosomal recessive) |
| SPP1 | Secreted phosphoprotein 1 |
| TGFBI | Transforming growth factor, beta-induced, 68kDa |
| THBS1 | Thrombospondin 1 |
| THBS2 | Thrombospondin 2 |
| THBS3 | Thrombospondin 3 |
| TIMP1 | Tissue inhibitor of matrix metalloprote 1 |
| TIMP2 | Tissue inhibitor of matrix metalloprote 2 |
| TIMP3 | Tissue inhibitor of matrix metalloproteinase 3 |
| CLEC3B | C-type lectin domain family 3, member B |
| TNC | Tenascin C |
| VCAM1 | Vascular cell adhesion molecule 1 |
| VTN | Vitronectin |
